# Pre-radiosurgery leucocyte ratios and modified glasgow prognostic score predict survival in non-small cell lung cancer brain metastases patients

**DOI:** 10.1007/s11060-020-03660-z

**Published:** 2020-11-11

**Authors:** Anna Cho, Helena Untersteiner, Dorian Hirschmann, Fabian Fitschek, Christian Dorfer, Karl Rössler, Sabine Zöchbauer-Müller, Brigitte Gatterbauer, Maximilian J. Hochmair, Josa M. Frischer

**Affiliations:** 1 Department of Neurosurgery, Medical University of Vienna, Waehringer Guertel 18-20, 1090 Vienna, Austria; 2 Division of Oncology, Department of Internal Medicine I, Medical University Vienna, Vienna, Austria; 3 Department of Respiratory and Critical Care Medicine, Karl Landsteiner Institute of Lung Research and Pulmonary Oncology, Vienna North Hospital, Vienna, Austria

**Keywords:** Gamma knife radiosurgery, Brain metastases, Neutrophil-to-lymphocyte ratio, Platelet-to-lymphocyte ratio, Lymphocyte-to-monocyte ratio, Modified glasgow prognostic score

## Abstract

**Introduction:**

The predictive value of the pre-radiosurgery Neutrophil-to-Lymphocyte Ratio (NLR), Platelet-to-Lymphocyte Ratio (PLR), Lymphocyte-to-Monocyte Ratio (LMR) and the modified Glasgow Prognostic Score (mGPS) was assessed for the first time in a homogenous group of NSCLC brain metastaes (BM) patients.

**Methods:**

We retrospectively evaluated 185 NSCLC-BM patients, who were treated with Gamma Knife Radiosurgery (GKRS). Patients with immunotherapy or targeted therapy were excluded. Routine laboratory parameters were reviewed within 14 days before GKRS1.

**Results:**

Median survival after GKRS1 was significantly longer in patients with NLR < 5 (p < 0.001), PLR < 180 (p = 0.003) and LMR ≥ 4 (p = 0.023). The Cox regression model for the continuous metric values revealed that each increase in the NLR of 1 equaled an increase of 4.3% in risk of death (HR: 1.043; 95%CI = 1.020–1.067, p < 0.001); each increase in the PLR of 10 caused an increase of 1.3% in risk of death (HR: 1.013; 95%CI = 1.004–1.021; p = 0.003) and each increase in the LMR of 1 equaled a decrease of 20.5% in risk of death (HR: 0.795; 95%CI = 0.697–0.907; p = 0.001). Moreover, the mGPS group was a highly significant predictor for survival after GKRS1 (p < 0.001) with a HR of 2.501 (95%CI = 1.582–3.954; p < 0.001). NLR, PLR, LMR values and mGPS groups were validated as independent prognostic factors for risk of death after adjusting for sex, KPS, age and presence of extracranial metastases.

**Conclusion:**

NLR, PLR, LMR and mGPS represent effective and simple tools to predict survival in NSCLC patients prior to radiosurgery for brain metastases.

## Introduction

Brain metastases (BM) are the most common intracranial tumors and occur in up to 60% of non-small-cell lung cancer (NSCLC) patients [1, 2]. Despite the improvement in primary cancer treatment, which led to a longer overall survival, the prognosis of patients with advanced lung cancer remains poor [3]. So far, different prognostic scores, such as the Graded Prognostic Assessment (GPA), Lung-molGPA, the Recursive Partitioning Analysis (RPA) and the Score Index for Radiosurgery (SIR), that incorporate multiple clinical parameters, have been used to predict the overall survival of BM patients [4–7].

In previous studies, several inflammatory parameters such as the Neutrophil-to-Lymphocyte Ratio (NLR), the Platelet-to-Lymphocyte Ratio (PLR), the Lymphocyte-to-Monocyte Ratio (LMR), but also the modified Glasgow Prognostic Score (mGPS), have been reported to be predictive for the overall survival in NSCLC patients [8–12]. We evaluated, for the first time, the prognostic value of pre-radiosurgery NLR, PLR, LMR and mGPS in a homogenous group of NSCLC patients with radiosurgically treated brain metastases.

## Material and methods

### Study population

**Fig. 1:**
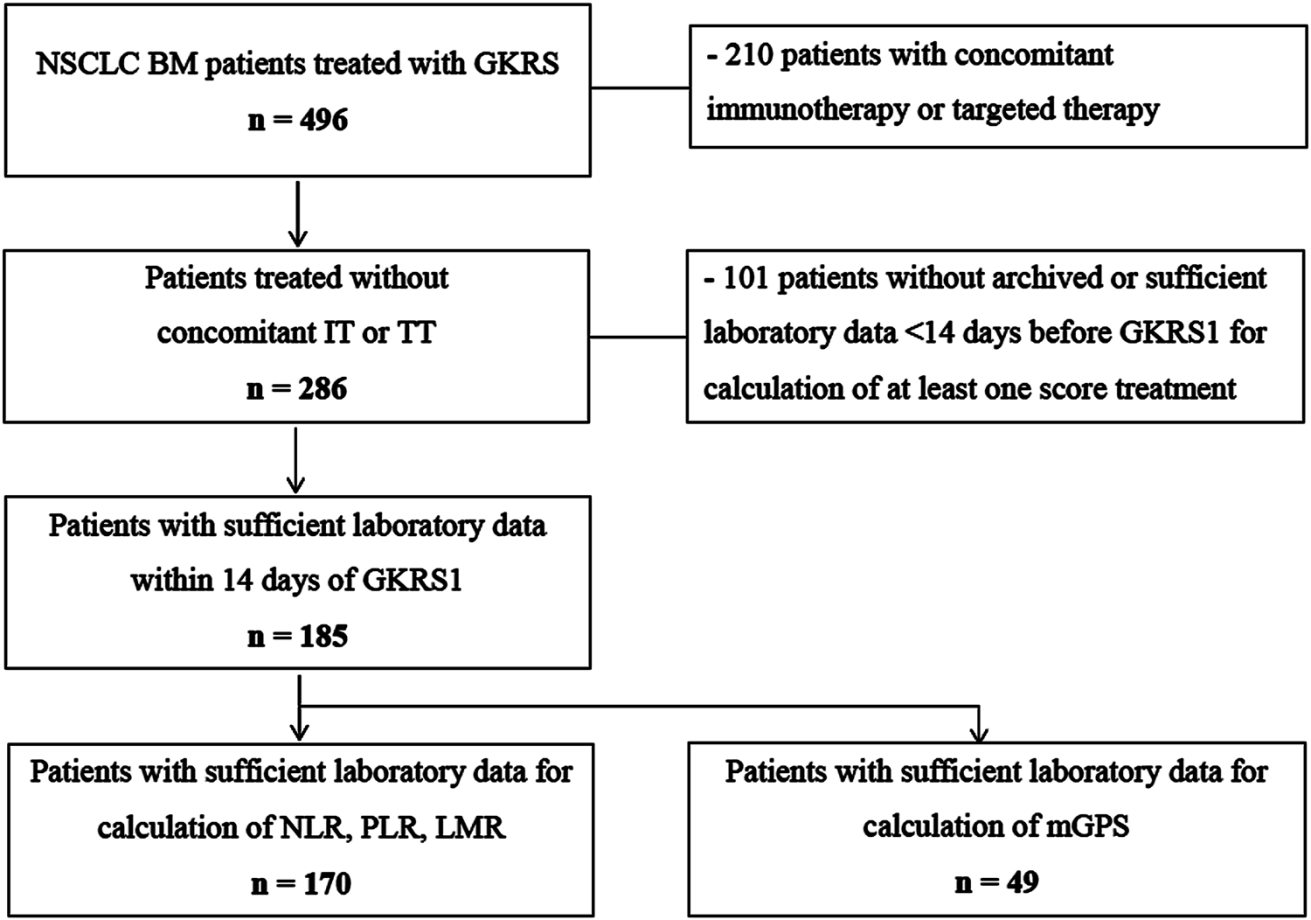
Flow chart depicting study inclusion algorithm. Between 2012 and 2018, 496 NSCLC patients with brain metastases (BM) were treated with GKRS. To evaluate the prognostic scores in a selected group of IT or TT naïve BM-NSCLC patients, 210 patients with concomitant immunotherapy or targeted therapy at (± 30 days) or after GKRS1 were excluded from the study. By excluding patients with previous or concomitant IT or TT, the prognostic scores could be evaluated in 286/496 (58%) patients. However, 101/286 (35%) patients without archived or sufficient laboratory data < 14 days before GKRS1 for calculation of at least one score had to be excluded. In total, 185/286 (65%) patients with sufficient laboratory data could be evaluated. Indeed, the baseline characterstics of the 185 included patients did not show any significant differences to the 101 excluded patients. The majority (170/185, 92%) of patients had available pre-treatment values for the calculation of NLR, PLR and LMR. However, due to missing albumin values, the mGPS could only be evaluated in 49/185 (26%) patients. *BM *brain metastases,* GKRS *Gamma Knife Radiosurgery,* IT *immunotherapy*, LMR *Lymphocyte-to-Monocyte-Ratio*, mGPS *modified Glasgow Prognostic Score*, NLR *Neutrophil-to-Lymphocyte Ratio*, NSCLC *non-small-cell lung cancer*, PLR *Platelet-to-Lymphocyte-Ratio*, TT *targeted therapy

At the Department of Neurosurgery, Medical University of Vienna, 496 NSCLC patients with BM were treated radiosurgically between 2012 and 2018, since the implementation of the new Gamma Knife® Perfexion™ in 2012. NSCLC-BM patients with an age > 18 years, at least one GKRS treatment for at least one BM and available laboratory data were retrospectively evaluated. Patients with concomitant immunotherapy or targeted therapy at (± 30 days) or after first Gamma Knife Radiosurgery (GKRS1) were excluded. Thus, 185 patients could be included in our study (Fig. 1; Table 1). The study complied with the Declaration of Helsinki and was approved by the local ethics review committee.

**Table 1:** Clinical characterization of the study population

	Time of first GKRS–total study population (n = 185)	Patients with available NLR, PLR and LMR (n = 170)	Patients with available mGPS (n = 49)
Age in years, median (range)	66 (36–87)	67 (36–87)	63 (49–84)
Female:male ratio	87:98	78:92	26:23
KPS in %, median (range)	80 (40–90)	80 (40–90)	80 (50–90)
KPS groups			
≥ 80%	113 (61%)	105 (62%)	25 (51%)
< 80%	72 (39%)	65 (38%)	24 (49%)
ECM Status at time of BM diagnosis			
Yes	110 (60%)	100 (59%)	32 (65%)
No	75 (40%)	70 (41%)	17 (35%)
NSCLC subtype			
Adenocarcinoma	142 (77%)	132 (78%)	41 (84%)
Squamous cell carcinoma	36 (20%)	31 (18%)	8 (16%)
Other	7 (3%)	7 (4%)	–
CNS treatment before GKRS1			
None	155 (84%)	141 (83%)	41 (84%)
WBRT and/or fRT	9 (5%)	9 (5%)	2 (4%)
BM resection without RT	7 (4%)	6 (4%)	3 (6%)
BM resection with WBRT and/or fRT	14 (7%)	14 (8%)	3 (6%)
Localization of BM at initial diagnosis			
Multiple	89 (48%)	85 (50%)	23 (47%)
Frontal	25 (14%)	22 (13%)	9 (19%)
Parietal	12 (7%)	11 (7%)	1 (2%)
Temporal	8 (4%)	8 (5%)	–
Occipital	10 (5%)	9 (5%)	3 (6%)
Central	12 (7%)	10 (6%)	2 (4%)
Basal ganglia/ brainstem /other	10 (5%)	9 (5%)	6 (12%)
Cerebellar	19 (10%)	16 (9%)	5 (10%)
Predicted survival after prognostic scores * in months, median (range)*			
GPA general	3.8 (2.6–11.0)	3.8 (2.6–11.0)	3.8 (2.6–11.0)
GPA specific	5.5 (3.0–14.8)	5.5 (3.0–14.8)	5.5 (3.0–14.8)
Lung-molGPA	13.7 (5.3–46.8)	13.7 (5.3–46.8)	13.7 (5.3–46.8)
RPA	4.5 (2.3–7.7)	4.5 (2.3–7.7)	4.5 (2.3–7.7)
SIR	6.0 (2.1–8.8)	6.0 (2.1–8.8)	6.0 (2.1–8.8)
New prognostic scores *in median (IQR)*			
NLR	5.4 (3.3–10.0)	5.4 (3.3–10.0)	6.6 (4.2–8.6)
PLR	202.6 (143.5–327.3)	202.6 (143.5–327.3)	211.5 (150.2–330.8)
LMR	2.0 (1.3–3.2)	2.0 (1.3–3.2)	2.2 (1.4–3.0)

Table 1 displays details of our 185 study patients at the time of first GKRS. NLR, PLR and LMR could be evaluated for 170/185 (92%) patients. Due to missing albumin values, the mGPS could only be reviewed for 49/185 (26%) patients. Prior CNS treatment was mainly performed for distant BM. At the time of BM diagnosis, the majority of patients had already been diagnosed with extracranial metastases. The overall survival was routinely evaluated by the Graded Prognostic Assessment (GPA general and specific), Lung-molGPA, recursive partitioning analysis (RPA) and the Score Index for Radiosurgery (SIR) for each patient. At the time of study conclusion, the majority of the patients (156/185, 84%) had already succumbed to their disease

*BM *brain metastases,* ECM *extracranial metastases,* fRT *fractionated radiotherapy,* GKRS *Gamma Knife Radiosurgery,* GPA *Graded Prognostic Assessment,* IQR *InterQuartile Range,* KPS *Karnofsky Performance Status Scale,* LMR *Lymphocyte-to-Monocyte Ratio,* mGPS *modified Glasgow Prognostic Score,* NLR *Neutrophil-to-Lymphocyte Ratio,* NSCLC *non-small-cell lung cancer,* PLR *Platelet-to-Lymphocyte Ratio,* RPA *recursive partitioning analysis,* SIR *Score Index for Radiosurgery,* WBRT *whole brain radiation therapy

### Laboratory parameters

Routine laboratory parameters, including white blood cell count, albumin, and CRP, were retrospectively reviewed within 14 days before GKRS1. To calculate the NLR, the absolute count of neutrophil granulocytes (G/L) was divided by the absolute count of lymphocytes (G/L). The PLR was calculated by dividing the platelet count (G/L) by the absolute count of lymphocytes (G/L). The absolute count of lymphocytes (G/L) was divided by the absolute count of monocytes (G/L) to calculate the LMR [11]. As previously published, the cut-off values were defined as 5 for NLR, 180 for PLR and 4 for LMR [11, 13, 14].

Furthermore, patients were classified as mGPS of 0 if normal CRP (≤ 10 mg/L) and albumin levels (≥ 35 g/L) could be observed. An mGPS of 1 was allocated if elevated CRP without hypoalbuminemia, i.e., CRP > 10 mg/l and albumin ≥ 35 g/L were diagnosed. If both parameters were altered, i.e., CRP > 10 mg/l and albumin < 35 g/L, the patients received an mGPS score of 2 [9].

### Radiosurgical technique

Patients were planned with GammaPlan and treated with Leksell Gamma Knife® Perfexion™ (Elekta AB, Stockholm, Sweden). The planning sequences were performed on a 1.5T magnet MRI, and always included Gadolinium contrast-enhanced T1-weighted MRI sequences in axial and coronal planes. The target was defined as a contrast-enhanced tumor mass on T1 sequences. The whole tumor mass was covered without an additional margin [15].

The median time between initial BM diagnosis and GKRS1 was 0.5 months (0.0–28.0). The majority of patients (137/185, 74%) underwent one GKRS, while 48/185 (26%) patients received multiple treatments due to newly diagnosed BM or two-fraction dose-staged GKRS as described before [16]. The median treatment volume was 0.6 cm^3^ (0.1–22.0). The prescribed doses mainly targeted the 50% (40–90) isodose line, with a median prescription dose of 18 Gy (8–20) and a median central dose of 30 Gy (12–44).

### Outcome evaluation and statistical analyses

After GKRS treatment, patients were routinely followed in a three-month interval by clinical and radiological assessment, according to our standard procedure. Furthermore, a death register comparison was performed for all patients.

Data were presented as counts and percentages or as median and range or interquartile range (IQR). Chi-square, Mann–Whitney U and Wilcoxon signed-rank tests were performed as appropriate. Median survival after GKRS1 was estimated by the Kaplan–Meier method and compared with the Log-Rank-Test. Shaffer correction was used after multiple comparisons. Univariate Cox proportional hazard regression analyses were applied to estimate the effect of each prognostic score on overall survival. Multivariate Cox regression analyses were performed including age group (≤ 65 vs. > 65a), sex, Karnofsky Performance Status (KPS) group (< 80% vs. ≥ 80%), presence of extracranial metastases (ECM) and NLR, PLR, LMR as continuous values or mGPS groups. For the validation of the proportional hazard assumption, the Log-Minus-Log plot function was applied. For all tests, p-values < 0.05 were considered to be statistically significant. Statistical analyses were carried out with IBM SPSS Statistics for Windows (Version 24 Armonk, NY: IBM Corp.).

## Results

### Patient characteristics and overall survival

The median time between the initial lung cancer diagnosis and the diagnosis of BM was 0.9 months (0.0–216.6). The estimated median overall survival time was 13.1 months [95% confidence interval (CI) = 9.3–16.9] after the initial diagnosis of NSCLC, 7.1 months (95% CI = 4.6–9.6) after the initial diagnosis of BMs, and 5.5 months (95% CI = 3.8–7.3) after GKRS1.

No significant difference in the survival after GKRS1 could be observed between female (87/185, 47%; 6.6 months; 95% CI = 3.3–10.0) and male patients (98/185, 53%; 4.8 months; 95% CI = 3.7–5.9; p = 0.080). Patients under 65 years (88/185, 48%; 7.5 months; 95% CI = 3.9–11.1) had a tendency towards a longer overall survival than patients over 65 years (97/185, 52%; 4.7 months; 95% CI = 3.1–6.2; p = 0.057).

In patients with a KPS of 80% or above (113/185, 61%), survival after GKRS1 was significantly longer (9.9 months, 95% CI = 6.5–13.3) than in patients with a KPS below 80% (72/185, 39%; 2.1 months, 95% CI = 1.2–2.9; p < 0.001). The estimated median survival after GKRS1 was longer in patients without ECM (75/185, 40%; 12.7 months; 95% CI = 10.4–14.9) than in patients with ECM (110/185, 60%; 4.6 months; 95% CI = 4.0–5.1; p < 0.001).

Furthermore, a significantly longer survival was observed in our study patients, compared to the calculated overall survival according to the general GPA (p < 0.001), specific GPA (p = 0.027), RPA (p < 0.001) and SIR (p = 0.001). In contrast, the Lung-molGPA score predicted a significantly longer overall survival (p < 0.001).

### Neutrophil-to-Lymphocyte ratio

**Fig. 2:**
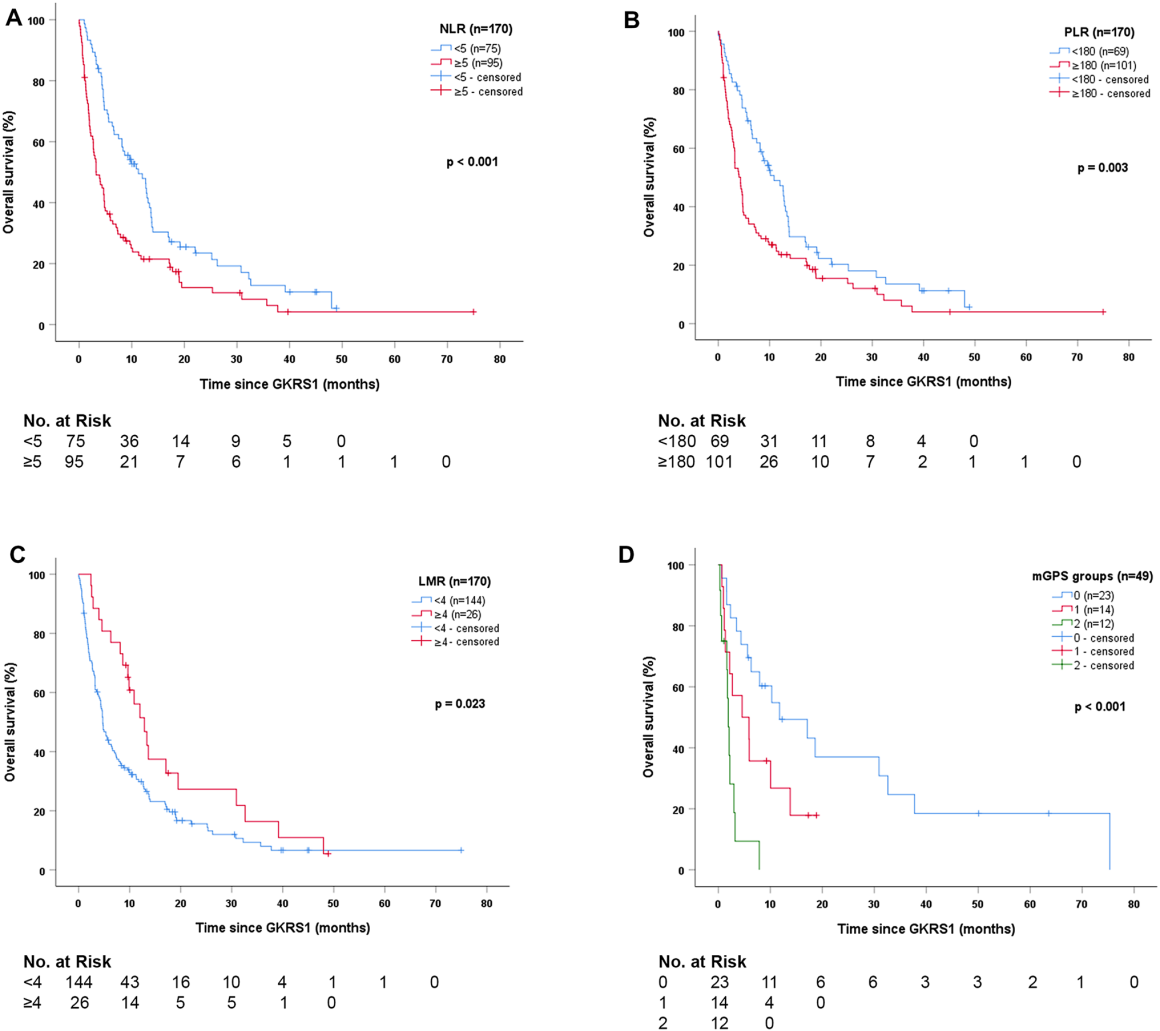
Survival after radiosurgery stratified according to prognostic scores. **a** The estimated survival after GKRS1 was significantly longer in patients with NLR < 5 (75/170, 44%; 11.3 months, 95% CI = 7.0–15.5) than in patients with NLR ≥ 5 (95/170, 56%; 3.3 months, 95% CI = 1.8–4.7; p < 0.001). **b** The estimated survival after GKRS1 was significantly longer in patients with PLR < 180 (69/170, 41%; 10.9 months, 95% CI = 6.9–14.8) than in patients with PLR ≥ 180 (101/170, 59%; 4.3 months, 95% CI = 3.1–5.6; p = 0.003). **c** The estimated survival after GKRS1 was significantly longer in patients with LMR ≥ 4 (26/170, 15%; 12.9 months, 95% CI = 9.3–16.5) than in patients with LMR < 4 (144/170, 85%; 4.7 months, 95% CI = 3.8–5.6, p = 0.023). **d** Patients with a mGPS score of 0 (23/49, 47%) showed the longest estimated median survival after GKRS1 with 11.8 months (95% CI = 0.2–23.3), followed by patients with mGPS of 1 (14/49, 29%) with 4.6 months (95% CI = 0.0–10.4), and mGPS of 2 (12/49, 24%) with 1.9 months (95% CI = 1.4–2.4; p < 0.001). Additional pairwise comparisons revealed significant differences in survival between mGPS scores 0 and 2 (p < 0.001) and between mGPS scores 1 and 2 (p = 0.022), even after Shaffer correction for multiple testing. Patients with a mGPS score of 0 only showed a tendency towards a longer survival after GKRS1 compared to patients with a mGPS score of 1 (p = 0.075). Thus, patients with poor mGPS scores, defined as mGPS 1 and 2, were pooled together. The comparison between mGPS score of 0 (23/49, 47%; 11.8 months; 95% CI = 0.2–23.3) and pooled mGPS score of 1 and 2 (26/49, 53%; 2.2 months; 95% CI = 1.2–3.2) additionally revealed significant differences in survival after GKRS1 (p = 0.002). *CI *confidence interval,* GKRS *Gamma Knife Radiosurgery,* LMR *Lymphocyte-to-Monocyte-Ratio*, mGPS *modified Glasgow Prognostic Score.* NLR *Neutrophil-to-Lymphocyte Ratio,* PLR *Platelet-to-Lymphocyte-Ratio

For the *Neutrophil-to-Lymphocyte Ratio*, the estimated median survial after GKRS1 was significantly longer in patients with NLR cut-off value of < 5 compared to patients with NLR ≥ 5 (p < 0.001, Fig. 2a). Consequently, the Cox regression model revealed a hazard ratio (HR) of 1.817 (95% CI = 1.301–2.539; p < 0.001).

Further, the Cox regression for the continuous metric NLR values showed that each increase in the NLR of 1 equaled an increase of 4.3% in risk of death (HR: 1.043; 95% CI = 1.020–1.067; p < 0.001).

The multivariate Cox regression model, including sex, KPS group, age group, presence of extracranial metastases and NLR as continuous values revealed rising NLR values (HR 1.028; 95% CI = 1.004–1.053; p = 0.022), KPS < 80% (HR: 2.574; 95% CI = 1.817–3.647; p < 0.001) and presence of ECM (HR: 1.854; 95% CI = 1.307–2.629; p = 0.001) as independent prognostic factors for an increased risk of death.

### Platelet-to-Lymphocyte ratio

For the *Platelet-to-Lymphocyte Ratio*, the estimated median survial after GKRS1 was significantly longer in patients with PLR cut-off value of < 180 compared to patients with PLR ≥ 180 (p = 0.003, Fig. 2b). Consequently, the Cox regression model displayed a HR of 1.654 (95% CI = 1.178–2.322; p = 0.004).

Further, the Cox regression for the continuous metric PLR values showed that each increase in the PLR of 10 equaled an increase of 1.3% in risk of death (HR: 1.013; 95% CI = 1.004–1.021; p = 0.003).

The multivariate Cox regression model, including sex, KPS group, age group, presence of extracranial metastases and PLR as continuous values revealed rising PLR values (HR: 1.009; 95% CI = 1.000–1.018; p = 0.046), KPS < 80% (HR: 2.723; 95% CI = 1.930–3.841; p < 0.001) and presence of ECM (HR: 1.803; 95% CI = 1.265–2.570; p = 0.001) as independent prognostic factors for an increased risk of death.

### Lymphocyte-to-Monocyte ratio

For the *Lymphocyte-to-Monocyte Ratio*, the estimated median survial after GKRS1 was significantly longer in patients with LMR cut-off value of ≥ 4 compared to patients with LMR < 4 (p = 0.023, Fig. 2c). Consequently, the Cox regression model displayed a HR of 0.585 (95% CI = 0.367–0.934; p = 0.025).

Further, the Cox regression for the continuous metric LMR values showed that each increase in the LMR of 1 equaled a decrease of 20.5% in risk of death (HR: 0.795; 95% CI = 0.697–0.907; p = 0.001).

The multivariate Cox regression model, including sex, KPS group, age group, presence of extracranial metastases and LMR as continuous values revealed KPS < 80% (HR: 2.541; 95% CI = 1.800–3.589; p < 0.001) and presence of ECM (HR: 1.856; 95% CI = 1.306–2.637; p = 0.001) as independent prognostic factors for an increased risk of death and rising LMR values (HR: 0.851; 95% CI = 0.748–0.968; p = 0.014) as an independent prognostic factor for a decreased risk of death.

### Modified Glasgow Prognostic score

The mGPS was 0 for 23/49 (47%), 1 for 14/49 (29%) and 2 for 12/49 (24%) patients. The estimated median overall survival showed significant differences along the mGPS groups. Patients with mGPS of 0 showed the longest estimated median overall survival, followed by patients with mGPS of 1 and mGPS of 2 (p < 0.001, Fig. 2d).

In addition, the Cox regression showed a HR of 2.501 (95% CI = 1.582–3.954; p < 0.001). The multivariate Cox regression model, including sex, KPS group, age group, presence of extracranial metastases and mGPS revealed a higher mGPS group (HR: 2.607; 95% CI = 1.634–4.160; p < 0.001) and KPS < 80% (HR: 2.236; 95% CI = 1.136–4.401; p = 0.020) as independent prognostic factors for an increased risk of death.

## Discussion

The ability to predict survival in lung cancer patients with simple and widely available prognostic scores is of importance and may help to facilitate clinical decision making and appropriate stratification of future clinical trials [7]. To our knowledge, we present the first study investigating the clinical relevance of pre-radiosurgery NLR, PLR, LMR and mGPS in a homogenous group of NSCLC patients with radiosurgically treated brain metastases.

So far, different prognostic scores, based on clinical and radiological characteristics, such as the general or specific GPA, RPA and SIR, have been applied to estimate the clinical outcome in patients with brain metastases [4–6]. However, the advent of modern oncological therapies has signifcantly improved the overall survival of lung cancer patients [3, 17, 18]. Moreover, recent publications reported on the prognostic value of genetic alterations in NSCLC patients [3, 7, 19]. Consequently, a new disease- and molecular-specific score, the Lung-molGPA, has been evaluated for its predictive value for overall survival in NSCLC patients by updating the original disease-specific GPA with the EGFR and ALK status [7].

However, as reported in the ESMO 2019 guidelines, there are several molecular drivers for oncogene addiction that represent strong predictive biomarkers and excellent therapeutic targets and thus, should be tested for [3, 19].

Our study population represents a homogenous group of IT or TT treatment naïve BM-NSCLC patients. By excluding patients with previous or concomitant immunotherapy or targeted therapy, the new prognostic scores, NLR, PLR, LMR and mGPS, could be validated in a patient cohort with similar baseline characteristics and thus, limited confounding factors. However, we might have selected patients who were not eligible for modern oncological therapies due to various reasons. Then again, our patient population displayed clinical baseline characteristics in line with previously published studies on radiosurgically treated BM patients, with the only exception of a slightly lower median KPS score [20]. The KPS alone is an important factor in the management of cancer patients and is included in various prognostic scores that we have additionally applied to our study cohort, such as the GPA, RPA, SIR and Lung-molGPA [4–7]. In our study, the observed survival was significantly longer compared to specific and general GPA, RPA or SIR but not compared to the Lung-molGPA score [4–7].

So far, NLR, PLR, LMR were recognized as significant prognostic indicators for survival in NSCLC patients at different time points of the disease, e.g. prior to general surgery, radiotherapy or oncological treatments [11, 21, 22]. The Neutrophil-to-Lymphocyte Ratio alone has also been identified as a prognostic factor for overall survival in patients with brain metastases of different primary tumors prior to microsurgical resection, prior to radiosurgery or within 30 days after radiosurgical treatment [3, 8, 23, 24].

In previous studies, different NLR, PLR, LMR cut-off values have been used to predict the overall survival in cancer patients. Thus, we applied previously reported cut-off values in NSCLC patients to our study population [11, 13]. In our study, NLR, PLR and LMR values evaluated prior to the first radiosurgical treatment, were predictive for survival after GKRS1. Consequently, we could validate the previously reported general NLR, PLR, and LMR cut-off values in our selected cohort of radiosurgically treated BM patients.

In addition to the easily applied cut-off values, the metric progression of each score was evaluated as significantly predictive. Each increase in the NLR of 1 lead to an increase of 4.3% in risk of death, and each increase in the PLR of 10 to an increase of 1.3% in risk of death. With each increase in the LMR of 1, a decrease of 20.5% in risk of death could be observed. Furthermore, these continous values of NLR, PLR and LMR were revealed as independent prognostic factors for risk of death even after adjusting for sex, KPS, age and presence of extracranial metastases. These findings might be explained by the association of inflammation and cancer progression [25]. The increase of platelets or neutrophils lead to the production of inflammatory cytokines and chemokines, causing tumor progression. The increase in neutrophils is also known to inhibit the cytolytic activity of lymphocytes, activated T cells and natural killer cells, but also to secrete tumor growth-promoting factors, leading to a tumor stimulating microenvironment. Therefore, neutrophilia is considered to worsen the prognosis [8, 23, 26]. Furthermore, lymphocytes are known to be essential for anti-tumor immunity. Thereby, the decrease of lymphocytes displays the impaired cell-mediated immune response [27]. Hence, leucocyte based ratios are considered as an indicator of inflammatory and tumor immune response, but also reflecting the extent of local leucocyte cell infiltration [27].

As another inflammatory index, decreased albumin levels represent a marker for malnutrition and systemic inflammation [28]. Moreover, increased CRP levels are known to be associated with lymphocytopenia, impaired T cell response within the tumor, and cancer progression [29]. Thus, the modified Glasgow Prognostic Score combines CRP and albumin values as a prognostic marker and has been validated in operable and inoperable NSCLC patients but not yet in patients with brain metastases [30, 31]. In our study, the mGPS group was highly predictive for survial after radiosurgery for BM, even after adjusting for sex, KPS, age and presence of extracranial metastases.

## Conclusions

The ability to predict survival in cancer patients with simple and widely available prognostic scores is of clinical importance. Still, at our institution, even patients in a palliative setting and multiple brain metastases are treated radiosurgically if any benefit from the treatment might be anticipated. Those decisions are always made according to the patients’ wishes and in the interdisciplinary agreement of the radiosurgeon and the oncologist. However, all four prognostic scores presented in this study, NLR, LMR, PLR and mGPS, represent simple tools to predict survival in NSCLC patients prior to radiosurgery for brain metastases and may help to facilitate patient counselling and appropriate stratification of future clinical trials.

## Limitations

Our limitations include its retrospective design and the related potential for selection bias due to the non-randomized treatment cohort. Although these four prognostic scores have been shown to be prognostic for survival in several studies in different patient populations and at different time points of the disease, the laboratory measurements at one single time point, as performed in our study, might be potentially biased.

## Data Availability

Research data will not be shared.

## Abbreviations

BM: Brain metastases
CI: Confidence Interval
CRP: C-reactive protein
ECM: Extracranial metastases
GKRS: Gamma Knife Radiosurgery
GPA: Graded Prognostic Assessment
HR: Hazard Ratio
IQR: Interquartile Range
IT: Immunotherapy
KPS: Karnofsky Perfomance Status Scale
LMR: Lymphocyte-to-Monocyte Ratio
mGPS: Modified Glasgow Prognostic Score
MRI: Magnetic resonance imaging
NLR: Neutrophil-to-Lymphocyte Ratio
NSCLC: Non-small-cell lung cancer
PLR: Platelet-to-Lymphocyte Ratio
RPA: Recursive partitioning analysis
SIR: Score Index for Radiosurgery
TT: Targeted therapy
WBRT: Whole-brain irradiation
